# Universal Strategy for Designing Shape Memory Hydrogels

**DOI:** 10.1021/acsmaterialslett.2c00107

**Published:** 2022-03-10

**Authors:** Dora C. S. Costa, Patrícia D. C. Costa, Maria C. Gomes, Amit Chandrakar, Paul A. Wieringa, Lorenzo Moroni, João F. Mano

**Affiliations:** † Department of Chemistry, CICECO—Aveiro Institute of Materials, University of Aveiro, Campus Universitário de Santiago, 3810-193 Aveiro, Portugal; ‡ MERLN Institute for Technology-Inspired Regenerative Medicine, Department of Complex Tissue Regeneration, Maastricht University, 6229 ER Maastricht, The Netherlands

## Abstract

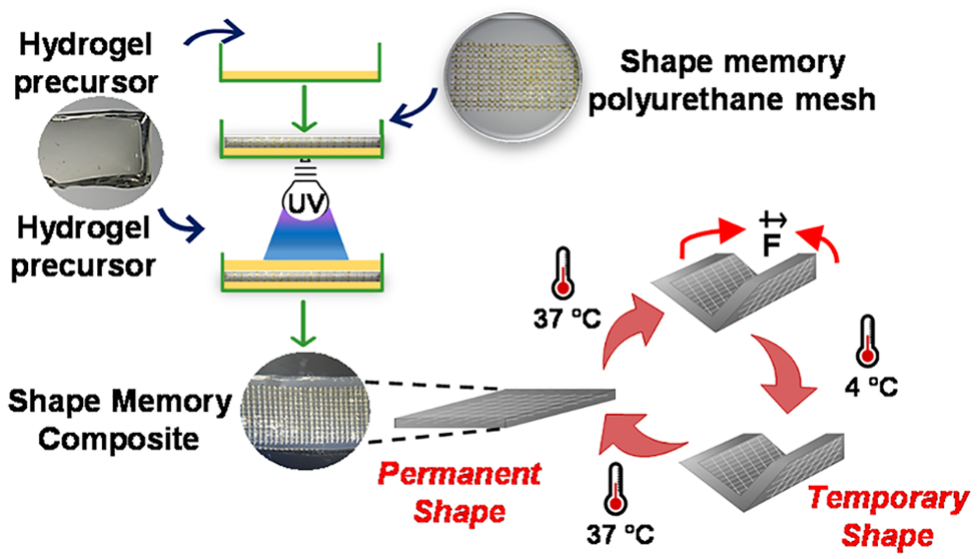

Smart polymeric biomaterials have been the focus of many recent biomedical studies, especially those with adaptability to defects and potential to be implanted in the human body. Herein we report a versatile and straightforward method to convert non-thermoresponsive hydrogels into thermoresponsive systems with shape memory ability. As a proof of concept, a thermoresponsive polyurethane mesh was embedded within a methacrylated chitosan (CHTMA), gelatin (GELMA), laminarin (LAMMA) or hyaluronic acid (HAMA) hydrogel network, which afforded hydrogel composites with shape memory ability. With this system, we achieved good to excellent shape fixity ratios (50–90%) and excellent shape recovery ratios (∼100%, almost instantaneously) at body temperature (37 °C). Cytocompatibility tests demonstrated good viability either with cells on top or encapsulated during all shape memory processes. This straightforward approach opens a broad range of possibilities to convey shape memory properties to virtually any synthetic or natural-based hydrogel for several biological and nonbiological applications.

Researchers have turned their attention to smart polymeric biomaterials and their potential use in medical devices, including structures for tissue engineering and regenerative medicine (TERM).^1^ Particularly, shape memory polymers (SMPs), which can undergo a predefined shape change in response to a given stimulus,^2,3^ have been explored. The ability of these materials to adapt to specific defect sites and potentially be implanted through minimally invasive strategies^4^ presents a considerable advantage for the development of advanced biomedical devices.^5−7^ Whenever possible, the use of natural polymers as building blocks to produce such materials allows taking advantage of their most beneficial properties, namely, biocompatibility, biodegradability, and ability to promote biological activity.^8,9^ However, most natural polymers do not possess stimuli-responsive features or shape memory capability under physiological conditions, even after chemical modifications are performed.^10^ This drawback has been largely surpassed by producing hybrid materials,^11^ in which natural polymers are combined with synthetic ones to integrate the biocompatibility properties of the former and the stimuli-responsive behavior of the latter.

Different stimuli can be employed to trigger shape memory behavior (e.g., light, pH, and humidity, among others).^9,12^ However, temperature-sensitive SMPs have attracted significant interest over a wide range of applications.^13^ Among the synthetic temperature-responsive polymers available for biomedical applications, polyurethanes have stood out because of their biocompatible and biodegradable character as well as their tunable and favorable mechanical properties.^14,15^ In addition, the transition temperature (*T*_trans_) of polyurethane (∼32 °C) is close to human body temperature,^16^ unlike those of other temperature-responsive polymers.^13^ Recently, Hendrikson et al.^17^ employed a shape memory polyurethane (SMPU) network to build scaffolds with 4D functionality, i.e., that change their shape when exposed to a given stimulus. The authors tested the temperature-induced shape memory ability of the developed scaffolds and evaluated how this process impacted cells seeded onto this material, concluding that the shape recovery process influenced the cell morphology.^17^

Inspired by this study, we conjectured whether this SMPU network could be used to impart shape memory ability onto any hydrogel, particularly when the hydrogel precursors do not possess stimuli-responsiveness properties. We hypothesized that when a SMPU structure, for example in the form of a mesh, is embedded within a non-thermoresponsive hydrogel network, the SMPU would allow the surrounding hydrogel to follow its movement upon shape fixity and recovery, conveying the shape memory property to the overall composite (Figure 1). To the best of our knowledge, this is a new approach for designing shape memory hydrogels with a universal tone, broadening the spectrum of polymers employed to build shape memory biomaterials. In this sense, to demonstrate the concept, we used methacrylated polymers such as methacrylated chitosan (CHTMA), methacrylated laminarin (LAMMA), and methacrylated hyaluronic acid (HAMA), which do not show temperature-induced shape memory behavior as precursor biomaterials for the hydrogel component. We also evaluated the temperature-responsive performance of methacrylated gelatin (GELMA) in this system. These polymers were chosen as simple and widely known platforms with described cytocompatibility^18−22^ (Figure 1).

**Figure 1:**
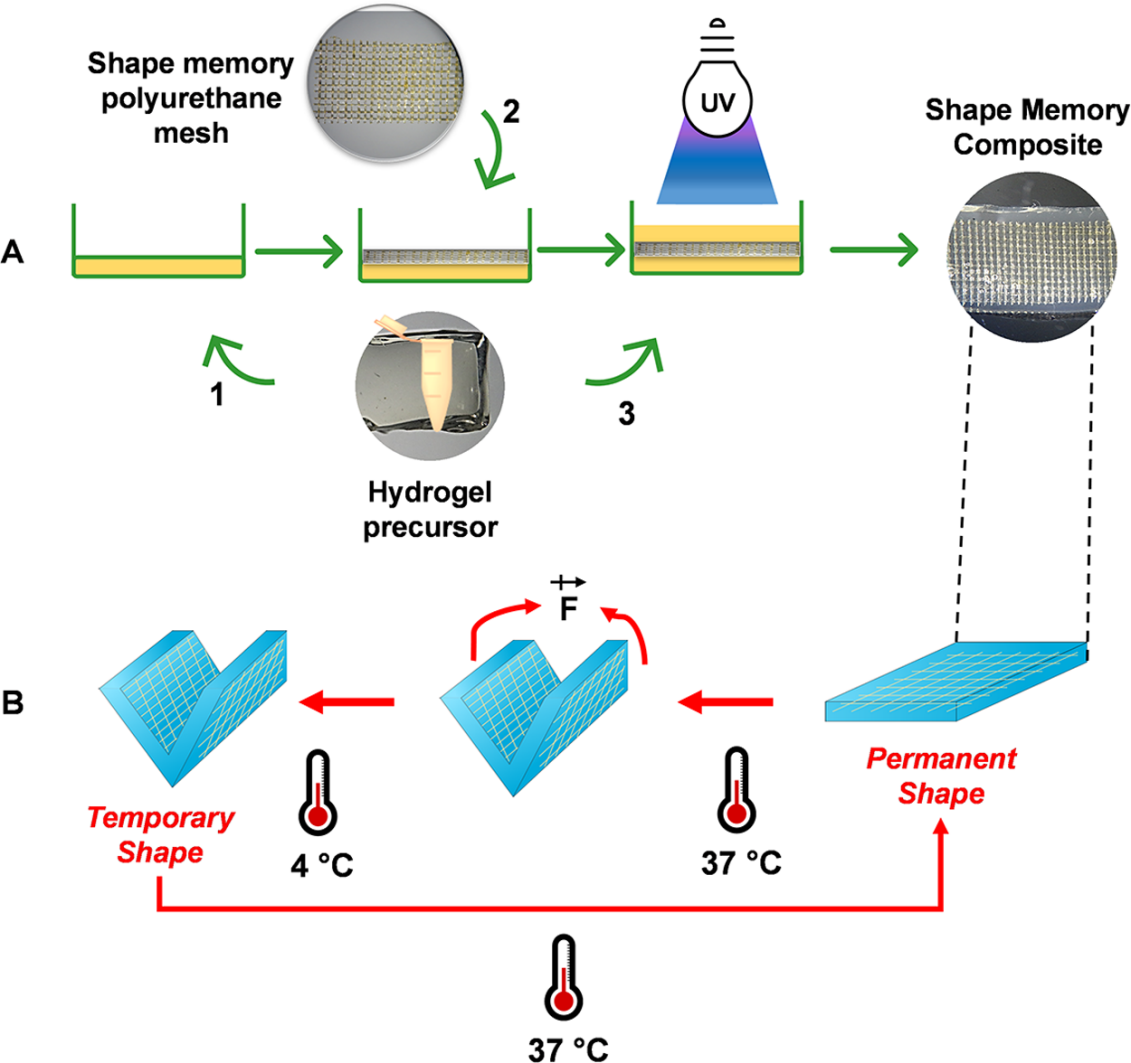
Schematic representations of (A) the preparation of polymer–SMPU composites and (B) their shape memory behavior.

With this, we propose a simple and broad range strategy for fabricating composites capable of adapting to specific defects, including hydrogels populated with cells either by encapsulation or a cell-seeding process. Initially, we prepared the non-thermoresponsive naturally derived materials. For that, CHTMA, GELMA, LAMMA, and HAMA were synthesized by coupling of chitosan (CHT), gelatin (GEL), laminarin (LAM), and hyaluronic acid (HA), respectively, with methacrylic acid, methacrylic anhydride, or glycidyl methacrylate on the basis of previous procedures.^18,21−23^ CHTMA, GELMA, LAMMA, and HAMA were obtained as white solids with a degree of substitution (DS) of 33%, 52%, 18%, and 11% or 29%, respectively. The insertion of methacrylate moieties onto the polymer backbones was confirmed and quantified by ^1^H NMR or UV–vis spectroscopy analysis (Figures S1–S5). The ^1^H NMR spectra showed two singlets at around 6.00 ppm, corresponding to the characteristic hydrogens H_a_ and H_b_ of the inserted methacrylic moieties, and a singlet at around 2.00 ppm, corresponding to the methyl hydrogens H_e_ of the inserted methacrylate moiety, which were in good agreement with previously reported data.^21−23^

SMPU meshes with a square pore size of 500 μm were fabricated via the melt electrowriting technique from SMPU pellets with *M*_w_ = 77 800 Da, *M*_n_ = 43 000 Da, PDI = 1.80, and a glass transition temperature of 35 °C (according to the SMP Technologies datasheet). The fiber diameter of the SMPU meshes was 58.6 ± 3.7 μm, resulting in pores that were 456.3 ± 23 μm in size.

To impart the hydrogel with shape memory capability, we envisioned the general pathway depicted in Figures 1, 2A, and S6. A CHTMA, GELMA, LAMMA, or HAMA hydrogel strip [a 5% w/v solution in cell culture medium (α-MEM)] was covalently photo-cross-linked (by exposure to UV light) with an embedded SMPU mesh (19.0 mm × 4.0 mm × 0.55 mm) in its network, corresponding to the initial/permanent shape of the polymer–SMPU composite. The composites were then exposed to a temperature of 65 or 37 °C to mold better and deform the SMPU mesh, and a temporary “U” or “L” shape was created by the application of an external force, followed by the fixation process at 4 °C for 6 h (Figures 2A and S6). The shape fixity ratio (*R*_f_) was then analyzed to determine the ability to fix the desired temporary form (Figure 2A). Subsequently, the sample was placed in preheated (37 °C) cell culture medium to promote the recovery of the permanent shape (Figures 2A and S6), and the shape recovery ratio (*R*_r_) was assessed to determine the ability of this composite to recover its original (permanent) form (Figures 2A and S6).

**Figure 2:**
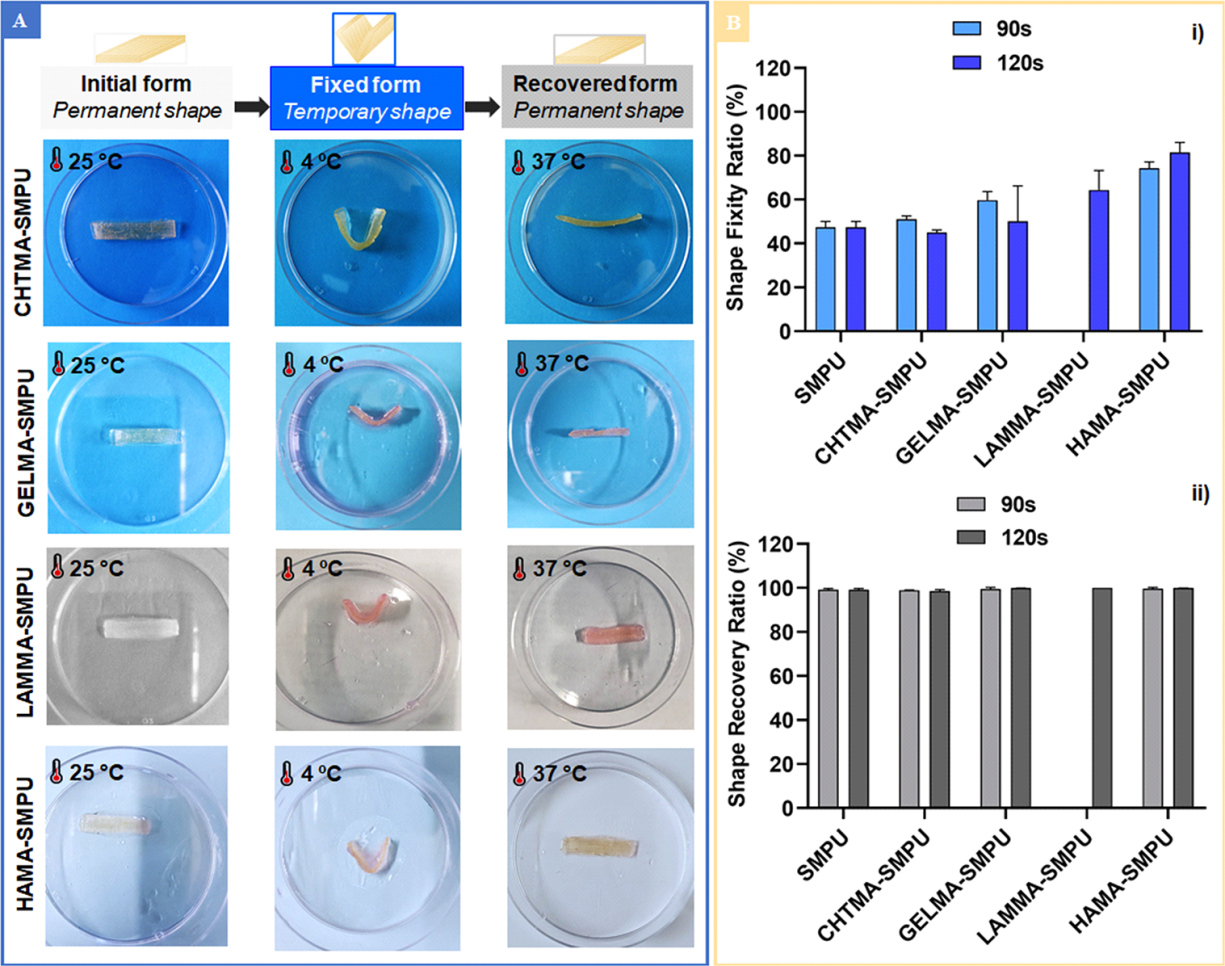
(A) Photographs showing the stages of the shape memory process of CHTMA–, GELMA–, LAMMA–, and HAMA–SMPU composites. (B) Comparison of SMPU and CHTMA–, GELMA–, LAMMA–, and HAMA–SMPU composites (with two different reticulation periods: 90 and 120 s): (i) shape fixity ratio (%) after a U-shaped deformation and (ii) shape recovery ratio (%) at 37 °C.

We decided to evaluate some parameters for each pathway step of our shape memory process. For the deformation step, we started our study by testing 65 °C (the same temperature used by Hendrikson et al.^17^) and then 37 °C using polymer–SMPU samples (20.0 mm × 5.0 mm × 3.0 mm) with different UV light exposure periods (90 s and 120s). The temperature (65 °C) did not affect the *R*_f_ results (data not shown), so we chose to move forward with 37 °C to recapitulate the physiological conditions better. The obtained *R*_f_ values for all conditions at 37 °C are compared in Figure 2B(i). The *R*_f_ values for the CHTMA–, GELMA–, and HAMA–SMPU composites were not significantly affected by changing the UV light exposure period. This could indicate almost complete cross-linking after the 90 s exposure. However, 90 s of UV light exposure was not enough for efficient cross-linking of the LAMMA network, maybe because of its low molecular weight compared with the other polymers. We observed an *R*_f_ range of 50–80%, with HAMA–SMPU achieving the highest *R*_f_ (∼80%) and CHTMA–SMPU showing the lowest *R*_f_ (∼50%). We also observed a 5–30% increasing *R*_f_ for the polymer–SMPU composites compared with pristine SMPU.

The value of *R*_f_ appears to depend on the hydrogel precursor stiffness, as shown in Figure 3 for the HAMA–SMPU composite. The *R*_f_ value tends to decrease with increasing DS (from 11 to 29%) and concentration (5 to 10% w/v) of the HAMA. This effect was greater for the DS parameter, resulting in a 20% *R*_f_ decrease.

**Figure 3:**
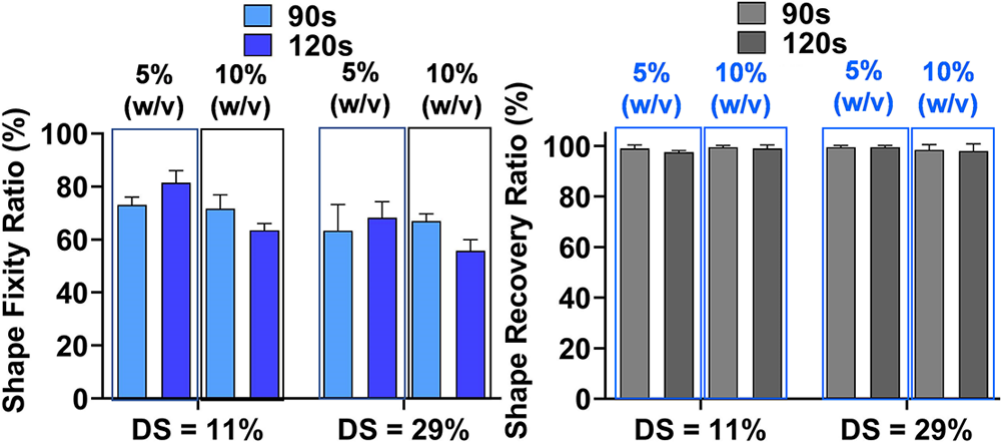
Effect of degree of substitution (DS) and concentration (% w/v) on (left) the shape fixity ratio and (right) the shape recovery ratio for the HAMA–SMPU composite (with two different reticulation periods: 90 and 120 s).

Another important parameter that could influence the *R*_f_ value is the bending angle of the deformation step. For example, the smaller the angle between the ends of the composite strip (θ_def_; see Table S1) is, the greater the applied force will be, which will reach the limit of the material. Our data support this fact: when we increased the deformation angle from ∼20° (Figure 2) to 90° (Figure 4) we achieved *R*_f_ ≈ 90%, which is an excellent result compared with other studies.^24^

**Figure 4:**
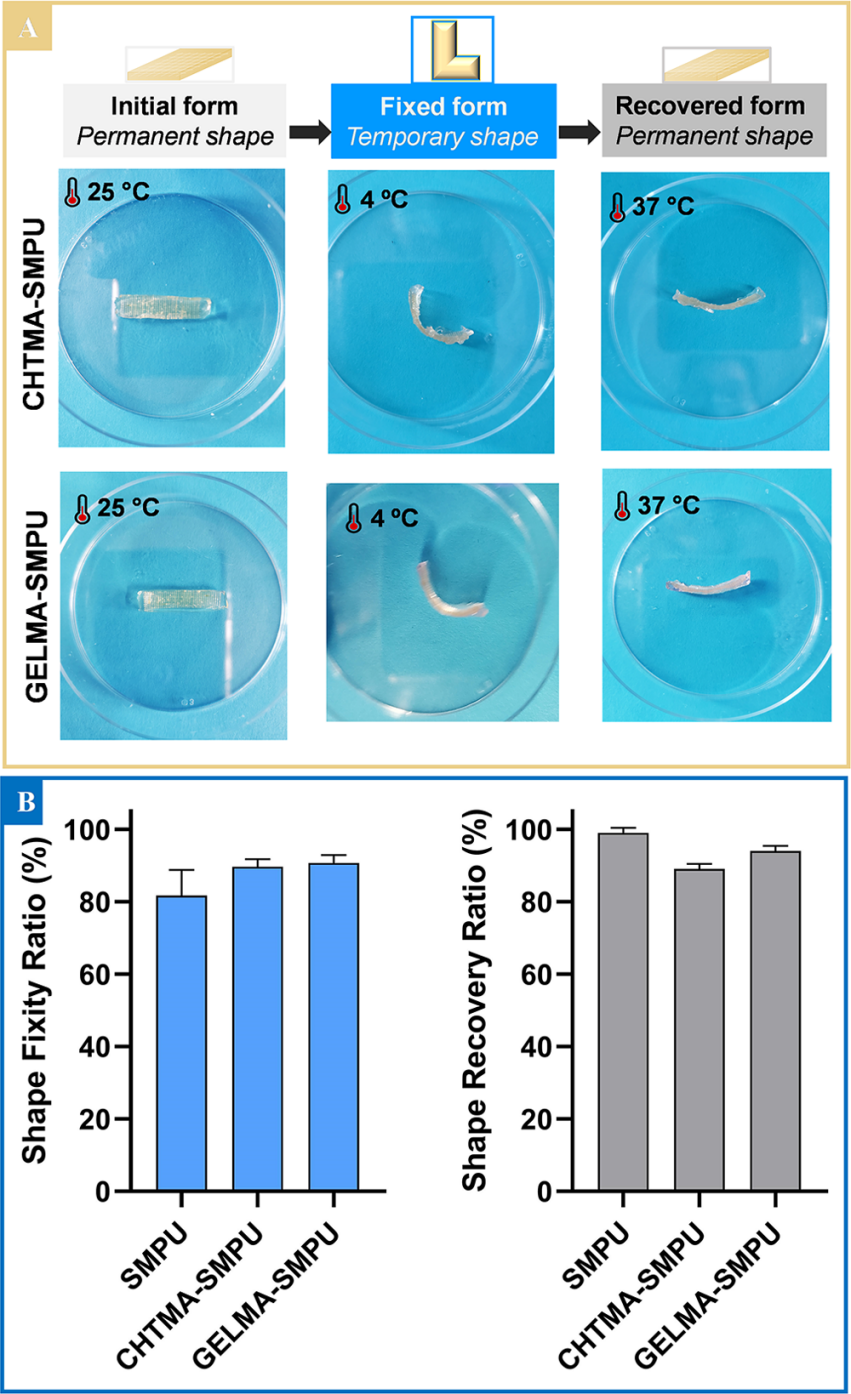
(A) Digital pictures of the stages of the shape memory process for a temporary L shape. (B) Shape fixity ratio (%) and shape recovery ratio (%) for CHTMA–SMPU and GELMA–SMPU composites.

The methacrylated polymer hydrogels themselves displayed *R*_f_ = 0% (the hydrogels broke into pieces in the deformation step) under all conditions, meaning that the SMPU mesh was responsible for the temperature-induced shape memory behavior of the material. The overall flexibility of the hydrogel matrix permits the deformation controlled by the embedded SMPU mesh. The composites can retain the shape memory behavior of the thin, pristine SMPU mesh.

With regard to the shape recovery step (i.e., the return to the permanent shape), we observed similar behavior under all conditions, consistently achieving *R*_r_ ≈ 100% after less than 2 min at 37 °C (Figure 2B(ii) and Video S1). The results support the universality of the system and clearly demonstrate that the hydrogels could fully accommodate the shape variation of the SMPU mesh. Therefore, the mechanism for our shape memory system is based on the well-studied thermoresponsive SMPU mechanism.^3,25−27^ Their structure composed by alternating disposition of hard segments (diisocyanate moieties) and soft segments (butanediol moieties) separated by urethane bonds allows them to switch between shapes as a result of the reversible motion between these segments, by working below and above their transition temperature (32 °C in our case).^17,26,27^

Targeting a material capable of adapting to a more realistic defect and being mechanically fixed to a cylindrical object, we conducted a shape memory study using a ring-shaped composite based on CHTMA–SMPU (Figure 5A). As can be observed, we were able to increase the diameter of the composite ring from 5 mm (permanent) to 7 mm (temporary) using an iron tube to apply an external force during the fixation step. With regard to the recovery step, after a few seconds at 37 °C, we observed the complete recovery to the initial diameter (Figure 5A).

**Figure 5:**
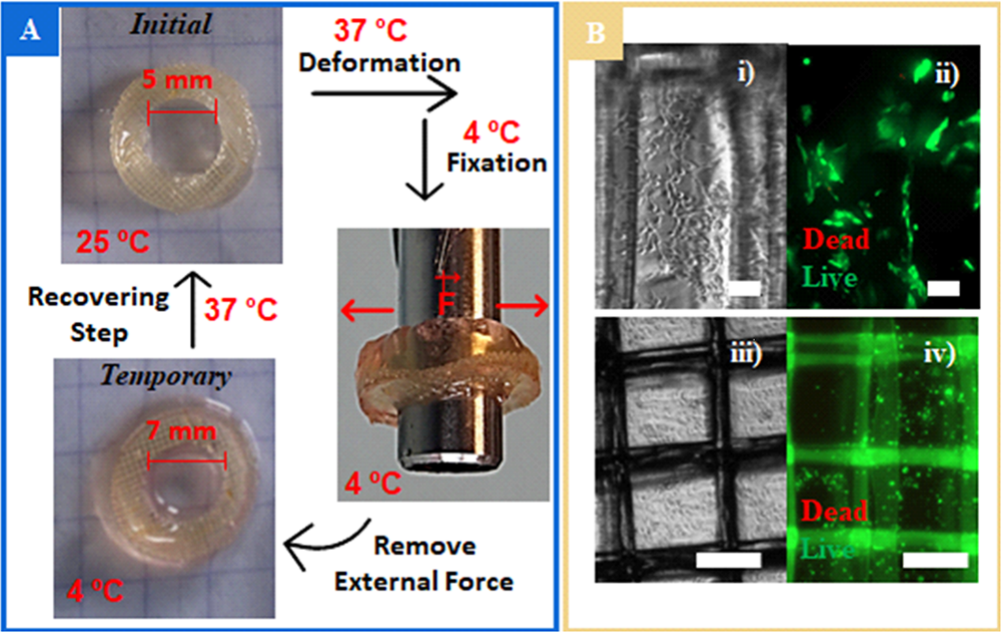
CHTMA–SMPU composite. (A) Digital images of ring-shaped deformation, fixation, and recovery steps. (B) Biocompatibility in MC3T3-E1 preosteoblasts: (i, ii) optical and fluorescence images, respectively, of cells seeded on the surface; (iii, iv) optical and fluorescence images of encapsulated cells after 1 day in culture. In the fluorescence images, live cells were stained with calcein-AM (green) and dead cells with propidium iodide (red). Scale bars: (i, ii) 100 μm; (iii, iv) 200 μm.

All of the polymers explored here have reported good cytocompatibility in many studies. We investigated whether the introduction of SMPU could elicit some cytotoxicity. Aiming to prove that the developed system gathered the necessary features for successful implantation, we evaluated the cytocompatibility for the case of the CHTMA–SMPU composite using MC3T3-E1 preosteoblasts as a cell model. On the basis of the beforementioned *R*_f_ results and envisioning cytocompatible conditions, we chose to conduct these studies at 37 °C. As depicted in Figure 5B(i, ii), preosteoblast cells seeded on top of the CHTMA–SMPU composite remained viable (as evidenced by the green staining) and were able to spread throughout the surface. This behavior indicates that the entire process to which the composite was submitted had no impact on the already known cytocompatibility of CHTMA.^20^ The possibility of cell encapsulation was assessed by entrapping cells in the CHTMA matrix alongside the SMPU mesh (Figure 5B(iii)) and subjecting them to the previously described shape fixation process using 37 °C as the deformation temperature. The encapsulated cells remained viable 1 day after encapsulation (Figure 5B(iv)), showing endurance to the entire shape memory process.

A previous report validated the cytocompatible nature of the SMPU mesh alone, which required a collagen coating of the SMPU surface for successful cell adhesion.^17^ In our case, the inclusion of the CHTMA hydrogel allowed us to suppress that requirement, enabling the creation of a symbiotic composite combining good cytocompatibility and shape memory behavior.

Although the fixation rate still needs some improvement, with these results we envisioned that this system could potentially be applied in several fields such as biomedical devices for TERM (e.g., smart valves,^28^ bone,^29^ cartilage,^30^ neural regeneration,^31^ and cardiac patches^32^ or vascular stents^33^), drug delivery,^34^ soft robotics/actuators, and 3D/4D (bio)printing.^3,9,11^

In summary, a novel and versatile approach to convey shape memory properties to virtually any synthetic or natural-based hydrogel was achieved, opening a path to new biomaterials that intrinsically do not have this ability. On the other hand, this strategy may also be used to bestow temperature-responsiveness to a hydrogel that already displays responsive behavior to other stimuli (pH or light, among others), conveying multiple shape changes on a single material triggered by different stimuli. The shape memory element introduced in the hydrogel was a mesh prepared by melt electrowriting, but we could envisage structures with more complex architectures that could be assembled with various types of hydrogels. The cytocompatibility test also supports that this system can be used for medical devices in TERM.

## Supporting Information Available

Materials, experimental procedures for polymer synthesis, all ^1^H NMR spectra, polymer–SMPU composite fabrication, shape memory studies, and biological assays (PDF)
Video showing shape recovery behavior (MP4)

## Abbreviations

CHT: chitosan
CHTMA: methacrylated chitosan
CHTMA–SMPU: methacrylated chitosan-shape memory polyurethane
GEL: gelatin
GELMA: methacrylated gelatin
GELMA–SMPU: methacrylated gelatin-shape memory polyurethane
HA: hyaluronic acid
HAMA: methacrylated hyaluronic acid
HAMA–SMPU: methacrylated hyaluronic acid-shape memory polyurethane
LAM: laminarin
LAMMA: methacrylated laminarin
LAMMA–SMPU: methacrylated laminarin-shape memory polyurethane
NMR: nuclear magnetic resonance
*R*_f_: shape fixity ratio
*R*_r_: shape recovery ratio
SMP: shape memory polymers
SMPU: shape memory polyurethane
TERM: tissue engineering and regenerative medicine
*T*_trans_: transition temperature
UV–vis: ultraviolet–visible
α-MEM: minimum essential medium α-modification
