# Supplementary material for: Universal Strategy for Designing Shape Memory Hydrogels
Source: ACS Mater Lett. 2022 Mar 10;4(4):701–6. doi: 10.1021/acsmaterialslett.2c00107 (PMC9777886; doi:10.1021/acsmaterialslett.2c00107)
Supplement: Supplementary file 1 — tz2c00107_si_001.pdf [file tz2c00107_si_001.pdf]

Supporting Information for

Universal Strategy for Designing Shape Memory Hydrogels

Dora C.S. Costa[a,], Patrícia D.C. Costa[a], Maria C. Gomes[a], Amit Chandrakar[b], Paul A. Wieringa[b], Lorenzo Moroni[b], João F. Mano[a,*]*

[a]Department of Chemistry, CICECO – Aveiro Institute of Materials, University of Aveiro, Campus Universitário de Santiago, 3810-193, Aveiro, Portugal
[b]Maastricht University, MERLN Institute for Technology-Inspired Regenerative Medicine, Department of Complex Tissue Regeneration, 6229ER, Maastricht, The Netherlands

*E-mails: doracosta@ua.pt (Dora C. S. Costa) and jmano@ua.pt (João F. Mano)

General Procedures

Materials and Equipments

All chemicals were used without further purification. Chitosan [CHT, Mw = ~96 kDa and deacetylation degree (DA) of 97.24% (Batch: S170420-1234,)] was kindly provided by Primex ehf (Iceland). Methacrylic acid (>99.0%), glycidyl methacrylate (>95%), *N*-(3-dimethylaminopropyl)-*N′*-ethylcarbodiimide hydrochloride [EDC, ≥98.0% (AT)], *N*-Hydroxysuccinimide, (NHS, >98.0%) and deuterium oxide (99.8%) were purchased from TCI Chemicals. Acetic acid [glacial, 99-100% (PA)], Hydrochloric acid, [≥37% (PA)], sodium bicarbonate, sodium chloride (99.9%), phosphate buffer saline (PBS), gelatin (GEL) from porcine skin (gel strength 300, Type A), methacrylic anhydride (94%), *N*,*N*-Dimethylformamide [DMF, 99% (PA)], Deuterated dimethyl sulfoxide (DMSO-d_6, 99.9%) and 2-Hydroxy-4'-(2-hydroxyethoxy)-2-methylpropiophenone (Irgacure 2959) were purchased from Sigma Aldrich. Laminarin (LAM) and hyaluronic acid (HA) sodium salt (Mw ~80 - 100 kDa, >91%) were acquired from Carbosynth (U.K.). Triethylamine (TEA, 99%) was bought from Acros Organics. 4-(*N*,*N*-dimethylamino)pyridine (DMAP) was acquired from VWR (U.K.). For the dialysis, it was used a 3.5 kD MWCO dialysis tubing [45 mm Flat-width, 15 meters/roll (50 ft)] from Repligen. Minimum Essential Medium α-modification (α-MEM), Fetal Bovine Serum, Live/Dead Kit and trinitrobenzene sulfonic acid (TNBSA, 5% w/v methanol solution) were purchased from ThermoScientific (USA). Sodium dodecyl sulphate (SDS) was purchased from Nzytech. ^{1}H NMR spectrum were recorded using a Bruker Avance 300 spectrometer at 300.13, using D_2O or DMSO-d_6 as internal reference. The chemical shifts are expressed in δ (ppm) and the coupling constants (*J*) in Hz. Methacrylated polymers were crosslinked using a Omnicure-S2000 XLA apparatus (320-500 nm). Cell viability was recorded on a Zeiss Axio Imager M2.

Fabrication of the Shape Memory Polyurethane (SMPU)

SMPU meshes were fabricated using a commercially available MEW instrument (Spraybase, A-1204-0001-01D), and mesh design were conceptualized using SEL Program Generator software (AIA America, Inc.). Briefly, SMPU (DiAPLEX MM 3520, SMP Technologies Inc., Nagoya, Japan) pellets were loaded into a syringe and heated to 200 °C for 30 minutes before printing to remove any air bubbles from the polymer melt. The melted polymer was then extruded using a 25G nozzle and 0.25 bar pressure. The distance between the nozzle and the collector plate was maintained at 5 mm. A positive voltage of 3.30 kV was applied to the collector plate, and the nozzle was grounded. All the meshes were fabricated at a critical velocity of 25 mm s^{-1}.

Synthesis of the Methacrylated Chitosan (CHTMA), Gelatin (GELMA), Laminarin (LAMMA) and Hyaluronic Acid (HAMA)

Methacrylated chitosan (CHTMA) *Gelatin (GELMA), Laminarin (LAMMA) and Hyaluronic Acid (HAMA)* were synthesized via EDC/NHS coupling chemistry, based on already reported procedures[1-4], with some modifications.

<u>*CHTMA*</u>

In short, chitosan (2.0 g) was dissolved in an aqueous acetic acid solution (0.05 M, 500.0 mL). Then, methacrylic acid was added at a ratio of [methacrylic acid]/[chitosan] = 5, after which EDC and NHS were added in a ratio of [EDC]/[chitosan] = 1.5 and [NHS]/[EDC] = 1. The reaction mixture was stirred for 24 hours, at room temperature (rt). Then, the reaction mixture was dialyzed against 5 L of the following aqueous solutions: $NaHCO_3$ (0.05 M), HCl

(0.001 M), NaCl (0.1 M) and distilled water, over a period of 7 days. Finally, CHTMA solution was freeze-dried, and the sample was stored at 4 °C until it was used.

GELMA

Gelatin (2.0 g) was dissolved in PBS (20% w/v, 20.0 mL) at 50 °C, then, methacrylic anhydride (0.8 mL) was added. The reaction mixture was maintained under vigorously stirring during to 3 h at 50 °C. After that, the mixture was diluted two times and dialyzed against 5 L of distilled water at 40 °C for 7 days (changing water twice a day). The purified product was freeze-dried and stored at 4 °C until it was used.

LAMMA

Laminarin (1.0 g) was dissolved in of DMSO (10 mL) under a nitrogen atmosphere; to this solution DMAP (0.167 g). Then, glycidyl methacrylate (2.9 mmol) was added. The mixture was stirred at rt for 48 h. An equimolar amount HCl solution (37% v/v) to neutralize DMAP was added. The LAMMA solution was purified by dialysis against distilled water for 7 days. Then, it was freeze-dried and stored under dry atmosphere.

HAMA

Hyaluronic acid (1.0 g) was dissolved in a (1:1) solution of distilled water/DMF (100 mL). Then, TEA (17.5 mL) and glycidyl methacrylated (17.5 mL) were added subsequently. The mixture was left for 3 days under stirring at room temperature (rt). The mixture was dialyzed against 5 L of distilled water for 7 days (changing water three times a day). The purified product was freeze-dried and stored at 4 °C before was used.

Determination of the degree of substitution (DS) of CHTMA, LAMMA and HAMA

The insertion of methacrylate moieties onto the polymer backbones were confirmed and quantified by ^{1}H nuclear magnetic resonance (NMR) or UV-Vis spectroscopy analysis[1-3]. Each freeze-dried methacrylated sample was dissolved in deuterium oxide or DMSO-d_6 at a concentration of 2% w/v. The obtained ^{1}H NMR spectra are depicted in Figures S1-S5, which are in good agreement with previous reported literature[1-4]. The substitution degree of CHTMA, LAMMA and HAMA were determined by comparing the relative integration area of the signals corresponding to the characteristic hydrogens of the inserted methacrylic moieties H_e and H_f (two singlets: around 6.00), and the signals corresponding to the hydrogens of the polymer backbone. The ratio between the relative integration areas of these signals yielded a degree of substitution (DS) equal to 33%, 18% and 11% (and 29%) for CHTMA, GELMA, LAMMA and HAMA, respectively. (Figure S1-S5).

Determination of the degree of substitution (DS) of GELMA

The substitution degree (DS) of GELMA was quantified using TNBSA assay, a free amino groups determination method. Briefly, standard solutions of glycine (0–20 μg mL^{-1}), pristine gelatin and GelMA (150 μg mL^{-1}) and TNBSA (0.01% w/v) were freshly prepared in $NaHCO_3$ buffer (aq. 0.1 M, pH ~8.5). To each sample (0.5 mL), the solution of TNBSA (0.25 mL) was added. The resulted mixture was incubated for 2 h at 37 °C. After this period, 10% (w/v) SDS aqueous solution (0.250 mL) and 1 M HCl aqueous solution (0.125 mL) was added to each mixture and mixed-well. The calibration curve was obtained by linear regression of the absorbance at 335 nm as a function of the glycine concentration. Three independent assays were conducted for gelatin and GELMA samples. The subtraction of the GELMA value from the gelatin value obtained from the resulted calibration curve equation yielded a 47% of free amino groups, which indicated a DS equal to 52%.

Preparation of CHTMA-, GELMA-, LAMMA- and HAMA-SMPU composites

To prepare the CHTMA-, *GELMA-, LAMMA- and HAMA*-SMPU composites materials, the freeze dried methacrylated polymers were dissolved in cell culture medium (α-MEM, pH ~7.4) at a concentration of 5% w/v, with 0.5% w/v of photoinitiator (Irgacure 2959). Half of the prepared solution (75.0 μL) was pipetted into a PDMS mold with a rectangular shape (20.0 mm x 5.0 mm). Then, a SMPU mesh sample (20.0 mm x 4.0 mm x 0.55 mm) with a pore size of 500 x 500 μm, of roughly the same size as the mold, was placed on top of this solution and covered with its remaining volume (75.0 μL). Then, the sample was irradiated by UV light (320-50nm), at a distance of 4 cm and irradiation power of 2 W/cm^2, for 90 or 120 seconds, to yield a composite material (20.0 mm x 5.0 mm x 3.0 mm) comprised of a covalently crosslinked hydrogel with an embedded SMPU mesh in its network.

Assessment of the shape memory performance

In order to assess the shape memory ability of the produced composite material, a straight strip of CHTMA-, *GELMA-LAMMA- and HAMA*-SMPU composites samples, with a size of 20 x 5 mm, were exposed to a temperature of 65 °C or 37 °C for 10 minutes, to allow the mechanical deformation of the sample in a "U" or "L" shape – temporary shape (Figure S6). Each sample was immersed into cell culture medium (α-MEM, pH ~7.4) inside of a sealed petri dish, which was then put into a water bath at 65 °C or 37 °C. Subsequently, the sample was transferred into pre-cooled (4 °C) cell culture medium (α-MEM, pH ~7.4), placed at 4 °C and kept at this temperature in the fridge during 6 hours, while maintaining a paper clip as mechanical force to perform the sample deformation. This step aimed at fixing the temporary "U" or "L" shape. Afterwards, the paper clip was removed, and the sample was placed in cell culture medium (α-MEM, pH ~7.4) bath at 37 °C, to induce the shape recovery process (Figure S6). The sample was monitored until the initial permanent shape was recovered (Figure S6 & Video S1). The evaluation of the shape memory process was quantified by measuring the angles at each stage of the shape memory process [initial angle (180°), deformation angle (θ_{def}), fixed angle (θ_f) and recovered angle (θ_r)], followed by the determination of the shape fixity ratio (R_f) and shape recovery ratio (R_r) based on the already reported mathematical equations[5-7], presented in Table S1.

Evaluation of CHTMA-SMPU biocompatibility

Cell Culture: The biological performance of CHTMA-SMPU composite was assessed using the MC3T3-E1 mouse pre-osteoblast cell line (European Collection of Authenticated Cell Cultures). MC3T3-E1 cells (between passage 44 and 47) were cultured in α-MEM supplemented with 10% fetal bovine serum and 1% antibiotic/antimycotic. At ~80% confluency, cells were detached by using trypsin-EDTA at 37 °C and centrifuged at 300 g for 5 min. To evaluate the biocompatibility of the CHTMA-SMPU composite, cells were i) seeded onto the CHTMA-SMPU composite or ii) incorporated into the CHMA solution to a final cell density of $5x10^5$ and $1x10^6$, respectively. For the latter, the same procedure described for the preparation of the CHTMA-SMPU composite was followed (37 °C, wet conditions).

Live/Dead Assay: After 1 day, the viability of MC3T3-E1 cells were assessed. CHTMA-SMPU composites were incubated in a solution of 2 µL of calcein AM 4×10^{-3} M solution in DMSO and 1 µL of propidium iodide 1 mg mL^{-1} in 1000 µL of PBS at 37 °C during 30 min. After washing with PBS, hydrogels were examined using a fluorescence microscope.

Supplementary Figures

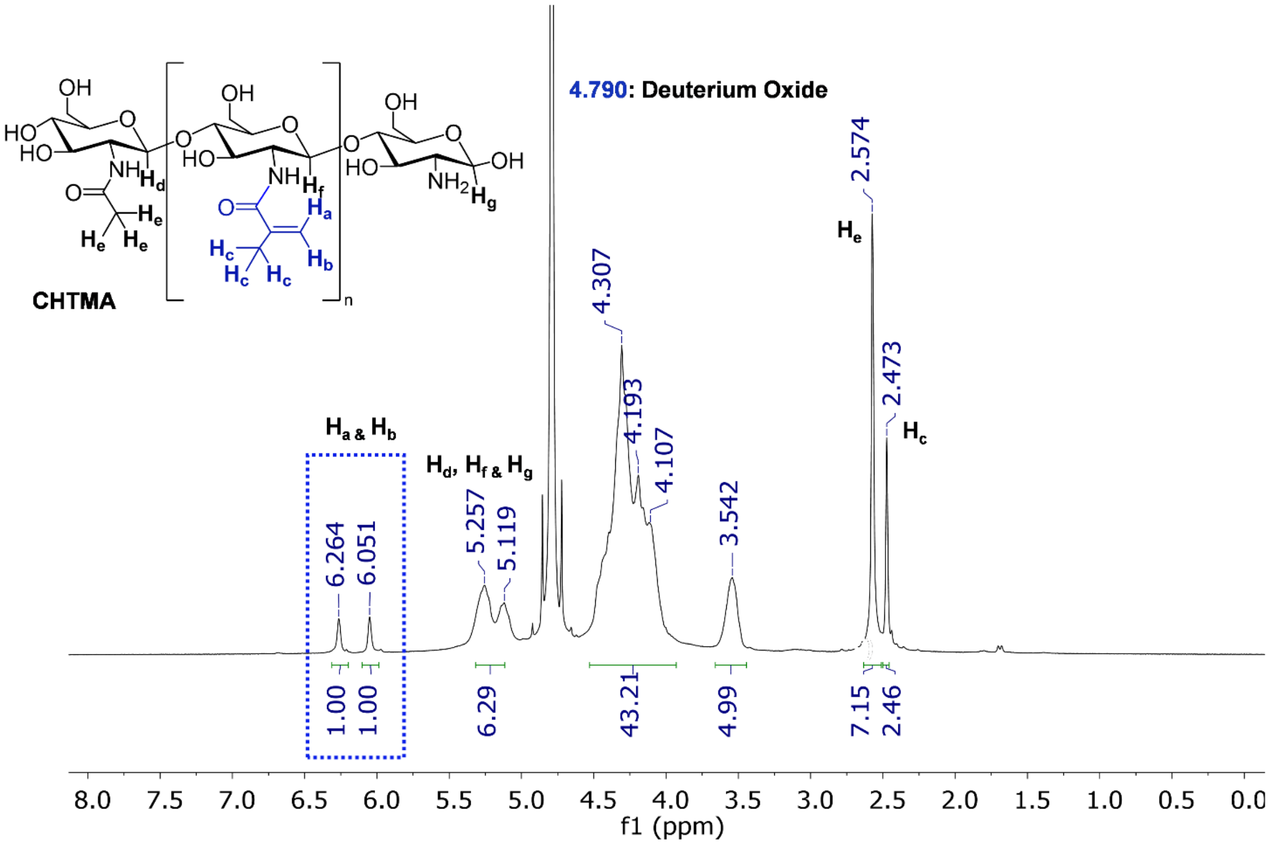

Figure S1. ^1H NMR spectrum (D_2O, 300.0 MHz, 70 °C, 512 scans) of CHTMA.

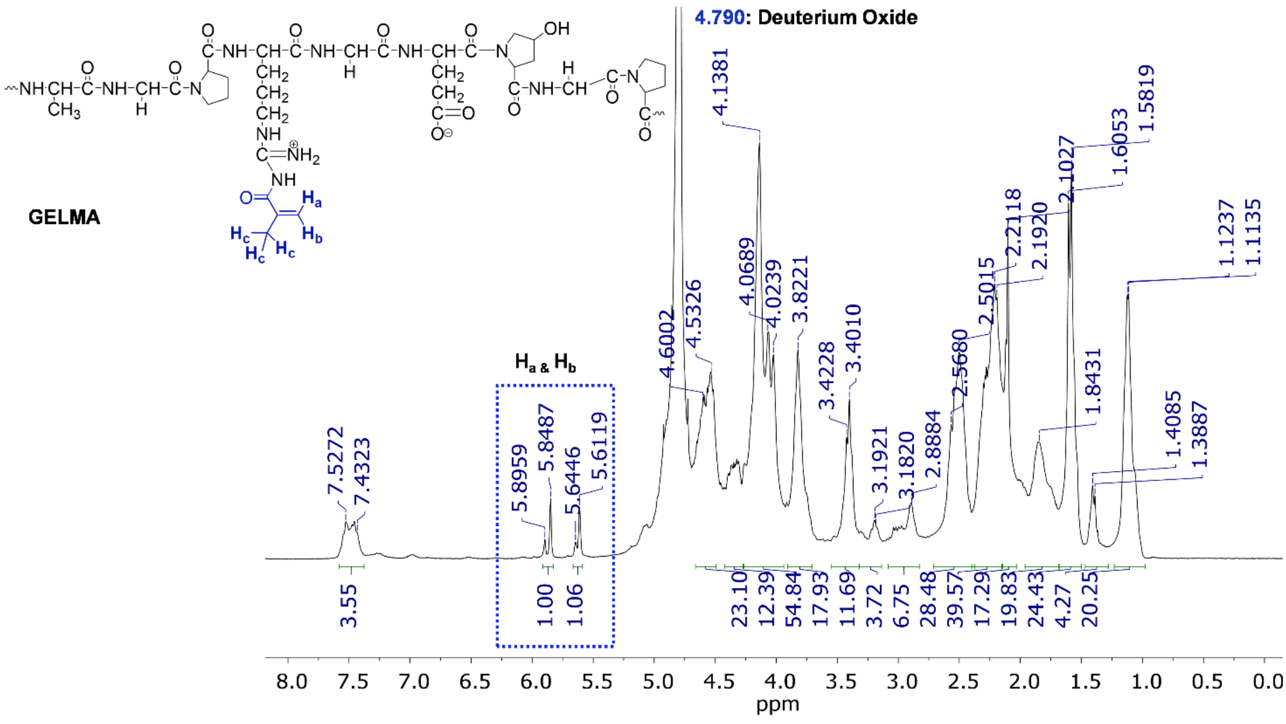

Figure S2. ^1H NMR spectrum (D_2O, 300.0 MHz, 40 °C, 512 scans) of GELMA.

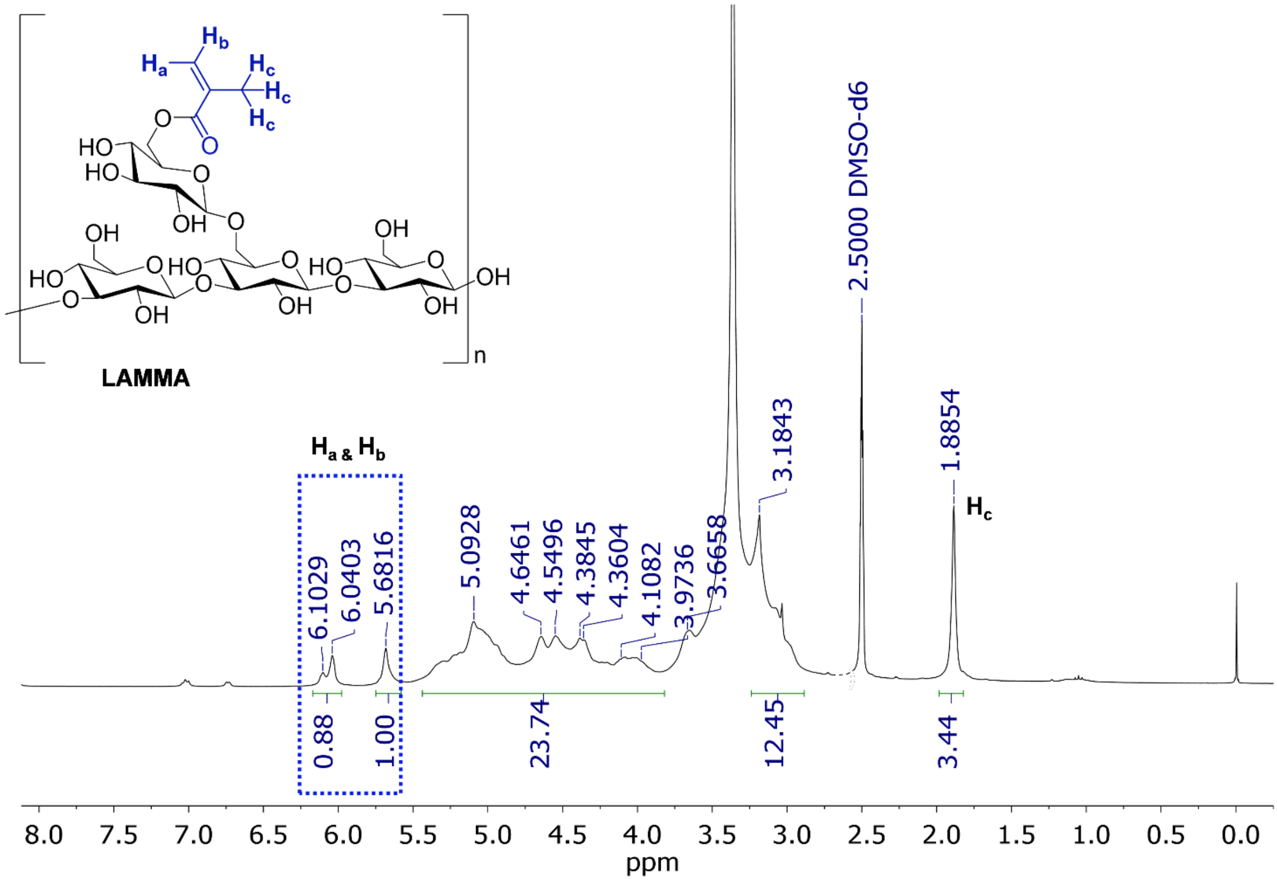

Figure S3. ^1H NMR spectrum (DMSO-d_6, 300.0 MHz, 512 scans) of LAMMA.

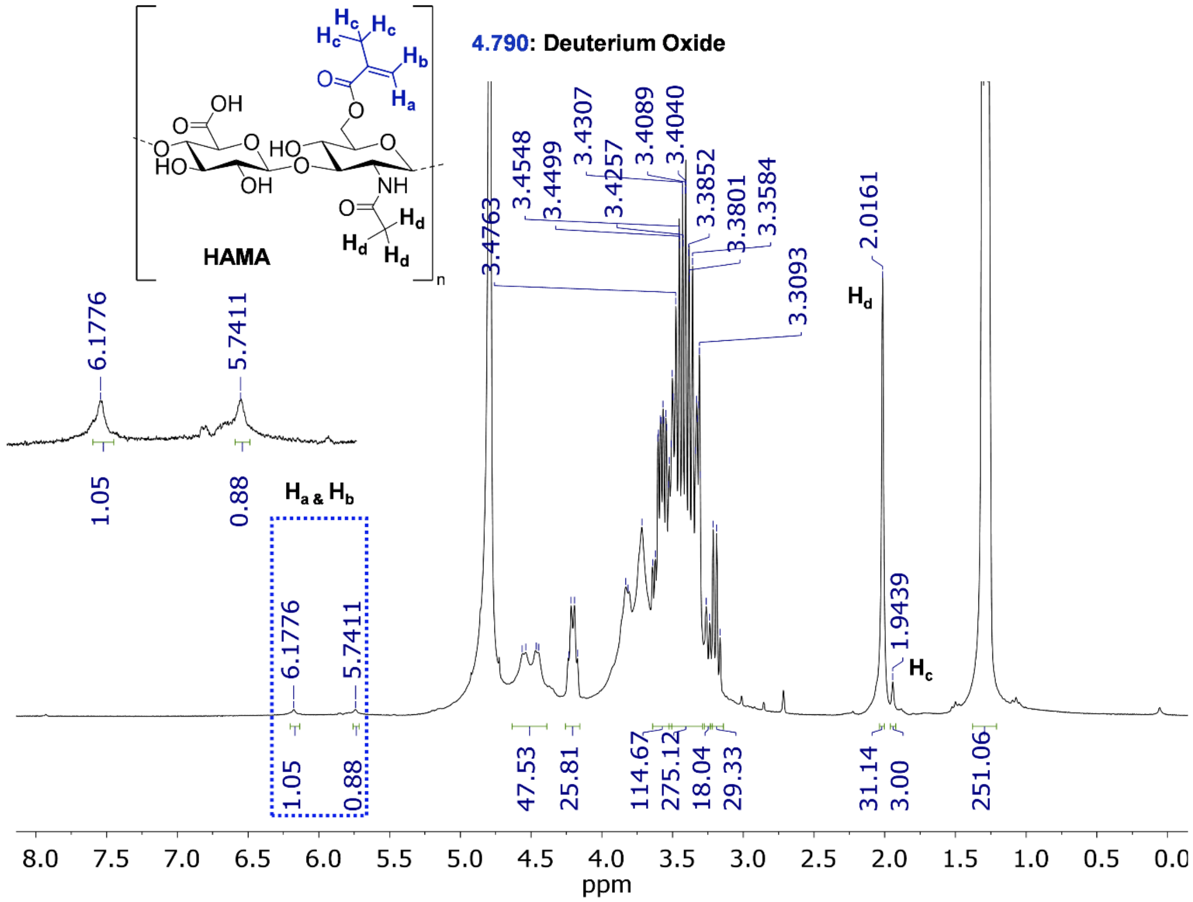

Figure S4. ^{1}H NMR spectrum (D_2O, 300.0 MHz, 512 scans) of HAMA with11% of DS.

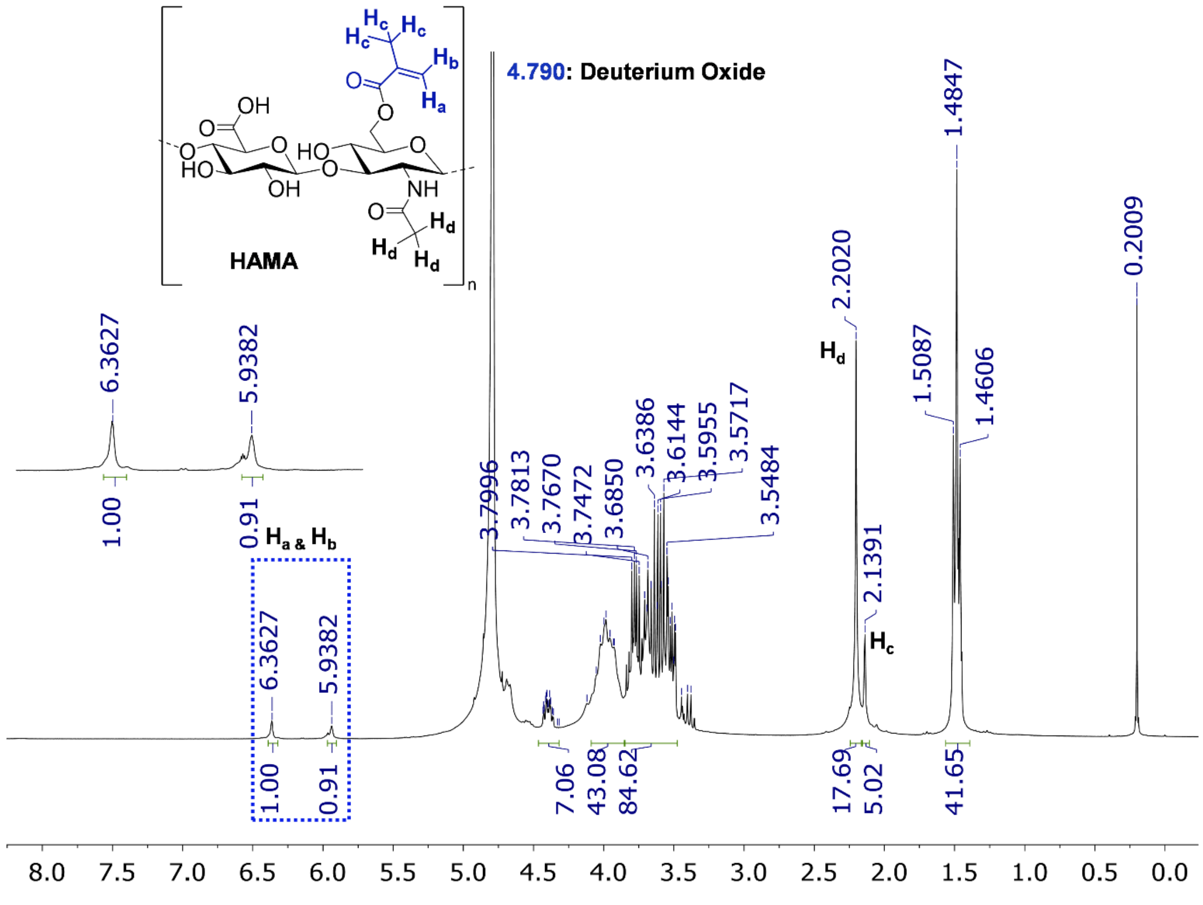

Figure S5. ^{1}H NMR spectrum (D_2O, 300.0 MHz, 512 scans) of HAMA with 29% of DS.

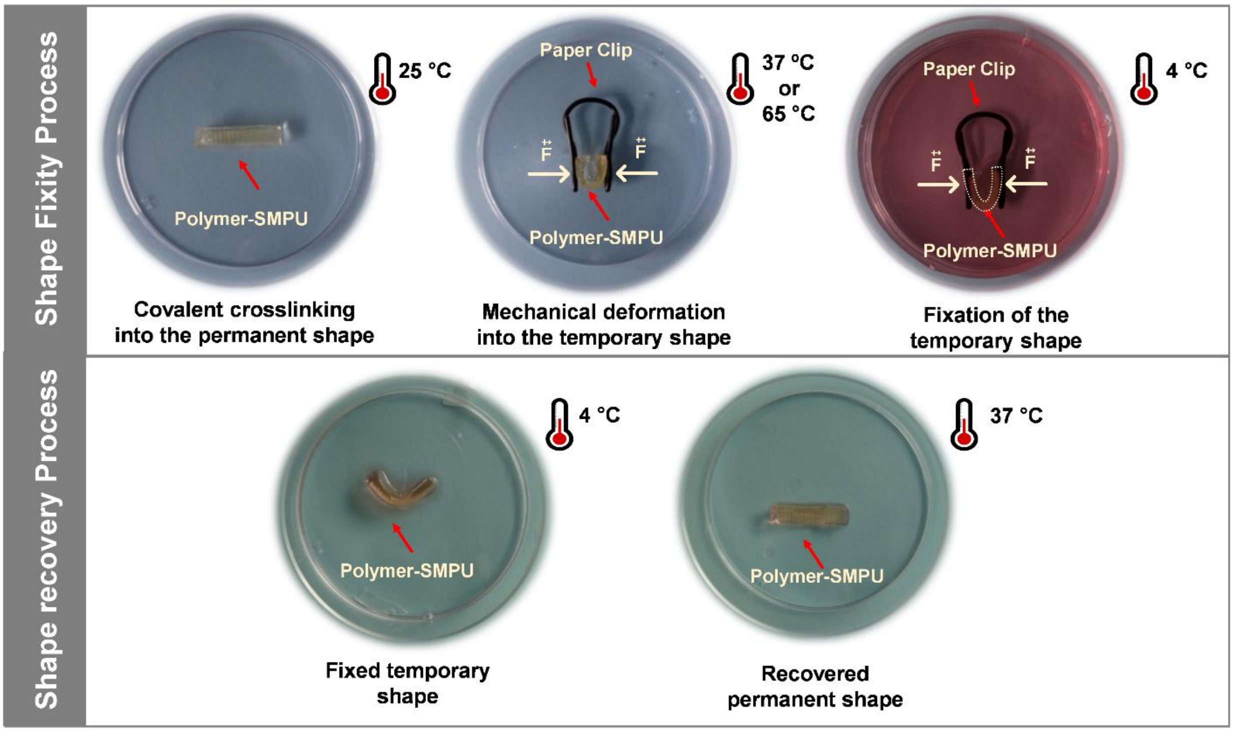

Figure S6. Showcase of Polymer-SMPU shape memory approach.

Table S1. Equation and variables description for shape fixity ratio (R_f) and shape recovery ratio (R_r) determination.

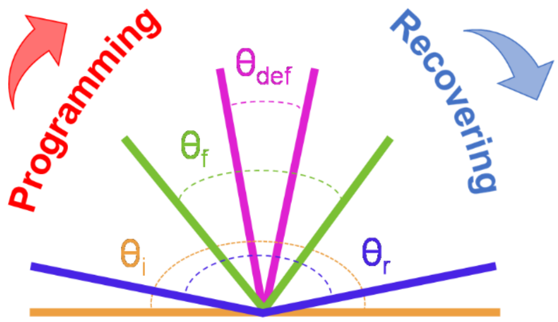	$R_f = \frac{\theta_i - \theta_f}{\theta_i - \theta_{def}} \times 100\%$ $R_r = \frac{\theta_r - \theta_f}{\theta_i - \theta_f} \times 100\%$
θ_i	**Initial angle:** angle value at the beginning of the programming stage (180°) (orange color).
θ_f	**Fixed angle**: angle value for the "U" or "L" shape at the end of the shape fixity stage, temporarily fixed angle (green color).
θ_{def}	**Deformation angle**: angle value of the paper clip used as mechanical force responsible for the sample deformation (pink color).
θ_r	**Recovered angle**: Angle value at the end of the shape recovery stage (purple color).

References

(1) Diolosa, M.; Donati, I.; Turco, G.; Cadenaro, M.; Di Lenarda, R.; Breschi, L.; Paoletti, S., Use of methacrylate-modified chitosan to increase the durability of dentine bonding systems. *Biomacromolecules* **2014,** *15*, 4606-13.

(2) Zhao, X.; Lang, Q.; Yildirimer, L.; Lin, Z. Y.; Cui, W.; Annabi, N.; Ng, K. W.; Dokmeci, M. R.; Ghaemmaghami, A. M.; Khademhosseini, A., Photocrosslinkable Gelatin Hydrogel for Epidermal Tissue Engineering. *Adv Healthc Mater* **2016,** *5*, 108-18.

(3) Custodio, C. A.; Reis, R. L.; Mano, J. F., Photo-Cross-Linked Laminarin-Based Hydrogels for Biomedical Applications. *Biomacromolecules* **2016,** *17*, 1602-9.

(4) A, S.; Xu, Q.; McMichael, P.; Gao, Y.; Li, X.; Wang, X.; Greiser, U.; Zhou, D.; Wang, W., A facile one-pot synthesis of acrylated hyaluronic acid. *Chem Commun (Camb)* **2018,** *54*, 1081-1084.

(5) Feng, W.; Zhou, W.; Zhang, S.; Fan, Y.; Yasin, A.; Yang, H., UV-controlled shape memory hydrogels triggered by photoacid generator. *RSC Advances* **2015,** *5*, 81784-81789.

(6) Khoury, L. R.; Popa, I., Chemical unfolding of protein domains induces shape change in programmed protein hydrogels. *Nat Commun* **2019,** *10*, 5439.

(7) Jiao, C.; Chen, Y.; Liu, T.; Peng, X.; Zhao, Y.; Zhang, J.; Wu, Y.; Wang, H., Rigid and Strong Thermoresponsive Shape Memory Hydrogels Transformed from Poly(vinylpyrrolidone- co-acryloxy acetophenone) Organogels. *ACS Appl Mater Interfaces* **2018,** *10*, 32707-32716.
